# Endogenous TRAIL-R4 critically impacts apoptotic and non-apoptotic TRAIL-induced signaling in cancer cells

**DOI:** 10.3389/fcell.2022.942718

**Published:** 2022-09-09

**Authors:** Anna-Christina Rambow, Insa Aschenbach, Sofie Hagelund, Doaa Tawfik, Jan-Paul Gundlach, Sebastian Weiße, Nicolai Maass, Anna Trauzold

**Affiliations:** ^1^ Department of Gynecology and Obstetrics, University Hospital Schleswig-Holstein (UKSH), Campus Kiel, Kiel, Germany; ^2^ Institute for Experimental Cancer Research, University of Kiel, Kiel, Germany; ^3^ Department of General Surgery, Visceral, Thoracic, Transplantation and Pediatric-Surgery, University Hospital Schleswig-Holstein (UKSH), Campus Kiel, Kiel, Germany

**Keywords:** TRAIL-R4, TRAIL, apoptosis, non-apoptotic signaling, pancreatic cancer, breast cancer, Bcl-xL

## Abstract

Binding of TRAIL to its death domain-containing receptors TRAIL-R1 and TRAIL-R2 can induce cell death and/or pro-inflammatory signaling. The importance of TRAIL and TRAIL-R1/R2 in tumor immune surveillance and cancer biology has meanwhile been well documented. In addition, TRAIL has been shown to preferentially kill tumor cells, raising hope for the development of targeted anti-cancer therapies. Apart from death-inducing receptors, TRAIL also binds to TRAIL-R3 and TRAIL-R4. Whereas TRAIL-R3 is lacking an intracellular domain entirely, TRAIL-R4 contains a truncated death domain but still a signaling-competent intracellular part. It is assumed that these receptors have anti-apoptotic, yet still not well understood regulatory functions.

To analyze the significance of the endogenous levels of TRAIL-R4 for TRAIL-induced signaling in cancer cells, we stably knocked down this receptor in Colo357 and MDA-MB-231 cells and analyzed the activation of apoptotic and non-apoptotic pathways in response to treatment with TRAIL.

We found that TRAIL-R4 affects a plethora of signaling pathways, partly in an opposite way. While knockdown of TRAIL-R4 in Colo357 strongly increased apoptosis and reduced clonogenic survival, it inhibited cell death and improved clonogenic survival of MDA-MB-231 cells after TRAIL treatment. Furthermore, TRAIL-R4 turned out to be an important regulator of the expression of a variety of anti-apoptotic proteins in MDA-MB-231 cells since TRAIL-R4-KD reduced the cellular levels of FLIPs, XIAP and cIAP2 but upregulated the levels of Bcl-xL. By inhibiting Bcl-xL with Navitoclax, we could finally show that this protein mainly accounts for the acquired resistance of MDA-MB-231 TRAIL-R4-KD cells to TRAIL-induced apoptosis. Analyses of non-apoptotic signaling pathways revealed that in both cell lines TRAIL-R4-KD resulted in a constitutively increased activity of AKT and ERK, while it reduced AKT activity after TRAIL treatment. Furthermore, TRAIL-R4-KD potentiated TRAIL-induced activation of ERK and p38 in Colo357, and NF-κB in MDA-MB-231 cells. Importantly, in both cell lines the activity of AKT, ERK, p38 and NF-κB after TRAIL treatment was higher in TRAIL-R4-KD cells than in respective control cells.

Thus, our data provide evidence for the important regulatory functions of endogenous TRAIL-R4 in cancer cells and improve our understanding of the very complex human TRAIL/TRAIL-R system.

## Introduction

Tumor necrosis factor-related apoptosis-inducing ligand (TRAIL) was discovered in 1995 (Wiley et al., 1995; Pitti et al., 1996) and soon thereafter it attracted huge interest due to its capability to induce cell death preferentially in cancer cells while sparing normal healthy cells (Ashkenazi et al., 1999; Walczak et al., 1999). It has been further recognized that it acts as an important player in the immune surveillance of tumors (Falschlehner et al., 2009). TRAIL exerts these functions by binding to its plasma membrane-expressed death receptors TRAIL-R1 and TRAIL-R2 which in their intracellular part possess a so-called death domain (DD) that is indispensable for cell death induction (Pan et al., 1997b; Sheridan et al., 1997; Walczak et al., 1997). The binding of TRAIL leads to receptor clustering and recruitment of Fas-associated Death Domain (FADD) to the DD of the receptors followed by recruitment of the initiator Caspase-8 and/or Caspase-10 (Schneider et al., 1997; Dickens et al., 2012). In the so formed death-inducing signaling complex (DISC) caspases become activated. The next steps of the apoptotic pathway depend on the cell type. In type I cells, activation of the initiator caspases is efficient enough to directly activate the effector Caspases-3, -6, and -7, which by cleavage of the plethora of their cellular targets execute cell death. In contrast, in type II cells, DISC formation is inefficient and the engagement of the mitochondrial apoptosis pathway is required to amplify the initial signal. In this case, Caspase-8 mediates the cleavage of the Bcl-2 homology (BH)3-only protein (Bid). Truncated Bid (tBid) in turn translocates to the mitochondria and activates the intrinsic apoptotic pathway. Oligomers of the Bcl-2 proteins, Bcl-2-associated X protein (Bax) and Bcl-2 antagonist or killer (Bak), cause mitochondrial outer membrane permeabilization (MOMP) (Wei et al., 2000; Kalkavan and Green, 2018). This leads to the release of Cytochrome c which together with the cytoplasmic apoptotic protease activating factor-1 (APAF1) protein and the initiator Caspase-9 builds the second platform for the activation of initiator caspases, the so-called apoptosome, in which Caspase-9 becomes activated (Pan et al., 1998b; Riedl and Salvesen, 2007). Activated Caspase-9, in turn, activates the downstream caspases Caspase-3, -6, and -7 eventually resulting in cell death.

The observation that TRAIL may specifically kill cancer cells led to the development of TRAIL-receptor-based therapeutics which were intensively studied in pre-clinical as well as clinical trials for a variety of tumor entities (von Karstedt et al., 2017). However, despite tremendous efforts, none of the compounds has met the expectations. One reason for these disappointing results is, among others, the constitutive and/or acquired resistance of cancer cells to TRAIL (Hanahan and Weinberg, 2000; Hinz et al., 2000; Trauzold et al., 2001; Trauzold et al., 2003).

The balance between cell death and cell survival is modulated by a multitude of regulatory proteins. Intracellularly, at the DISC level, c-FLIP can inhibit Caspase-8 thereby preventing apoptosis (Safa, 2013). In type II cells, the anti-apoptotic members of the Bcl-2 family, B-cell lymphoma 2 (Bcl-2) and B-cell lymphoma extra-large (Bcl-xL), control apoptosis by binding to the pro-apoptotic proteins Bax and Bak, thereby hindering MOMP (Kalkavan and Green, 2018). Last but not least, members of the Inhibitors of Apoptosis (IAP) proteins can bind to and inhibit the activity of apoptotic caspases (Silke and Meier, 2013). Within the apoptotic signal transduction pathway, permeabilization of the mitochondria leads to the release of a second mitochondria activator of caspases/direct IAP binding protein with low PI (SMAC/DIABLO) into the cytosol, which in turn binds to and inhibits the IAPs thereby ensuring an efficient activation of caspases (Lalaoui et al., 2020).

In addition, it has been realized that TRAIL death receptors, apart from inducing apoptosis, can also activate several pro-inflammatory signal transduction pathways like Mitogen-activated protein kinases (MAPKs), nuclear factor kappa-light-chain-enhancer of activated B-cells (NF-κB), Src kinase (Src), protein kinase C (PKC) and AKT which may enhance tumor cell malignancy (Degli-Esposti et al., 1997a; Schneider et al., 1997; Trauzold et al., 2001; Secchiero et al., 2003; Siegmund et al., 2007; Azijli et al., 2012; Azijli et al., 2013). Indeed, it has been demonstrated that TRAIL can promote tumor cell proliferation, migration, invasion and metastasis (Ishimura et al., 2006; Trauzold et al., 2006; Hoogwater et al., 2010; von Karstedt et al., 2015).

The TRAIL/TRAIL-R system in humans is highly complex. The ligand can exist in two forms, as a transmembrane protein and as a soluble protein cleaved from the transmembrane form by still not well understood proteolytic events (Mariani and Krammer, 1998). Also, TRAIL-R2 can exist in two forms as a result of alternative splicing of a single gene (Screaton et al., 1997). Besides the former mentioned apoptosis-inducing death receptors, TRAIL-R1 and TRAIL-R2, two other plasma membrane-expressed receptors, namely TRAIL-R3 and TRAIL-R4, exist, which most probably play a regulatory role (von Karstedt et al., 2017). Because of the lack of a functional DD, TRAIL-R3 and TRAIL-R4 are not able to induce cell death (Degli-Esposti et al., 1997b; Marsters et al., 1997). TRAIL-R3 does not contain any intracellular domain and is, therefore, unable to transmit TRAIL signals into the cell. However, it can “catch” TRAIL and is thus believed to act as a decoy receptor (MacFarlane et al., 1997). TRAIL-R4 possesses a long intracellular part but only a truncated, non-functional DD. It has been proposed that TRAIL-R4 can inhibit TRAIL-induced cell death by acting either as a decoy receptor and/or by affecting the proper DISC formation by clustering with the TRAIL death receptors (Degli-Esposti et al., 1997a; Marsters et al., 1997).

All TRAIL-Rs can form homo- and heterocomplexes, before and after ligand binding, providing the possibility of an extremely fine tuning of the TRAIL-induced response (Kischkel et al., 2000; Valley et al., 2012). This, however, represents another difficulty in understanding the role of the individual participants in the system. In fact, only the functions of TRAIL-R1 and TRAIL-R2 have been studied extensively due to their potential role as targets for apoptosis-inducing anti-cancer therapies, and meanwhile are pretty well understood. The functions of the other TRAIL-Rs, in particular of the signaling competent TRAIL-R4, still remain enigmatic.

Of note, some studies identified the overexpression of TRAIL-R4 in tumors, a fact that could be correlated to a more malignant phenotype and poor patients’ prognosis (Koksal et al., 2008; Ganten et al., 2009; Sanlioglu et al., 2009). On the other hand, some tumors down regulate the expression of TRAIL-R4 (van Noesel et al., 2002; Shivapurkar et al., 2004; Macher-Goeppinger et al., 2009), suggesting a context-dependent and/or cancer specific function of this receptor.

In our study, we aimed to elucidate the significance of the endogenous levels of TRAIL-R4 for TRAIL-induced signaling pathways in cancer cells. Therefore, we stably knocked down TRAIL-R4 in two different human cancer cell lines and analyzed the activation of apoptotic and non-apoptotic, pro-inflammatory signaling pathways in response to treatment with recombinant TRAIL. Our findings improve the understanding of the complex TRAIL/TRAIL-R functions and point to a critical regulatory role of TRAIL-R4 in this system.

## Materials and methods

### Cell Culture

The human PDAC cell line Colo357 and human breast cancer cell line MDA-MB-231 were cultured in RPMI-1640 medium (Sigma-Aldrich, Hamburg, Germany) supplemented with 1% Glutamine (Gibco, Darmstadt, Germany), 1% Sodium Pyruvate (Gibco) and 10% Fetal bovine serum (Pan BioTech, Aidenbach, Germany) under standard cell culture conditions (37°C, 5% CO_2_) up to 70–85% confluence. Cell culture supernatant was regularly tested to avoid mycoplasma contamination using Venor^®^ GeM classic mycoplasma detection kit (Minerva Biolabs GmbH, Berlin, Germany) according to the manufacturer’s protocol. Because of the higher TRAIL sensitivity of Colo357 compared to MDA-MB-231 cells, for stimulation experiments we used 50 ng/ml recombinant human sTRAIL/Apo2L (TRAIL; PeproTech, Hamburg, Germany) for Colo357 and 100 ng/ml for MDA-MB-231 cells, respectively. For inhibition of non-apoptotic signaling pathways, following agents at the indicated end concentrations were used prior to (pre-incubation for 2 h) and during TRAIL treatment: MEK1/2 inhibitor (U0126, Promega, Madison, Wisconsin, United States; 10 μM), JNK inhibitor (JNK-IN-8, Calbiochem, San Diego, California, United States; 1 μM), IKK2 inhibitor (IKK2 inhibitor VIII, Calbiochem; 10 μM), p38 inhibitor (SB203580, Calbiochem; 10 μM), Src inhibitor (Dasatinib, Selleck Chemicals, Houston, Texas, United States; 100 nM), AKT inhibitor (MK-2206, Selleck Chemicals; 1 μM), and the broad-spectrum inhibitor of apoptotic caspases zVAD-fmk (Bachem Holding, Bubendorf, Switzerland; 20 μM). To inhibit IAPs, the bivalent SMAC mimetic Birinapant (Selleck Chemicals), a potent antagonist of cIAP1, cIAP2 and XIAP was used at the concentration of 1 μM prior to (pre-incubation for 3–5 h) and during TRAIL treatment.

### Generating stable TRAIL-R4 knockdown cell lines

For stable knockdown of TRAIL-R4, Colo357 and MDA-MB-231 cells were transduced with the GIPZ Lentiviral Human TNFRSF10D shRNA (clone ID: V3LHS_344449 and V2LHS_239709) or with the non-silencing control vector and selected with 2 μg/ml puromycin. All vectors were purchased from Dharmacon, GE Healthcare, Lafayette, United States.

### Clonogenic survival assay

Cells were seeded at 1.000 cells per well in 6-well format and stimulated with 50 ng/ml (Colo357) or 100 ng/ml (MDA-MB-231) TRAIL, respectively. After 24 h cell culture medium was replaced by fresh medium without TRAIL and cells were incubated for additional 6 days to allow colony formation. Cells were visualized and colonies quantified by CELLAVISTA^®^ cell imager (SYNENTEC GmbH, Elmshorn, Germany) and finally stained with 0.5% crystal violet (in 20% MeOH) to visualize living cells and formed colonies. Crystal violet was thoroughly washed away with ddH_2_O and plates were left to dry.

### Cytotoxicity/viability assay

Cells were seeded at 1 × 10^4^ cells/well in 96-well format. After 24 h cells were stimulated with 50 ng/ml (Colo357) or 100 ng/ml (MDA-MB-231) TRAIL, respectively. 24 h later cell viability and cell death were assessed by triple-fluorescence staining with Propidium Iodide (PI) (10 μg/ml, Invitrogen, Karlsruhe, Germany), Calcein AM (1 μg/ml, BioLegend, San Diego, California, United States) and Hoechst 33342 (2.5 μg/ml, Sigma-Aldrich). PI/Calcein AM/Hoechst 33342 staining was performed by diluting the substances in PBS and adding to the wells at an end concentration of 1:100 PI, 1:10.000 Calcein AM, 1:1000 Hoechst 33342, respectively. To avoid loss of dead cells no washing steps or medium changes were performed. Cells were incubated with the staining solution in the dark at 37°C and 5% CO_2_ for 20min and subsequently cell fluorescence imaging was performed by CELLAVISTA^®^ cell imager. Pictures were obtained and quantified using YT-software^®^ (SYNENTEC GmbH): Cells stained with Hoechst 33342 and Calcein AM were considered living cells, whereas PI stained cells were counted as dead cells due to loss of membrane integrity.

### Crystal violet cell viability assay

Cells were seeded at 1 × 10^4^ cells per well in 96-well format. After 24 h cells were stimulated with 50 ng/ml (Colo357) or 100 ng/ml (MDA-MB-231) TRAIL for 24 h. In some experiments cells were incubated with either U0126, MK-2206 (see above), Birinapant (see above), Navitoclax or Venetoclax (both 5 μM, Selleck Chemicals) prior to (for 2 h) and concomitant with TRAIL (for 24 h). Cell viability was assayed by crystal violet staining as described previously (Trauzold et al., 2006).

### Western blotting

Cells were lysed in RIPA buffer supplemented with Complete Protease Inhibitor Cocktail and PhosphoStop (both from Roche, Mannheim, Germany). Protein lysates were separated by SDS-PAGE using Novex™ WedgeWell™ 4–20% Tris-Glycin Gels (Thermo Fisher Scientific, United States), transferred onto Polyvinylidene fluoride (PVDF-) membranes (Immobilon^®^-P Transfer Membrane, Merck Millipore, Darmstadt, Germany) and reactive proteins were detected with the following primary antibodies: Cell Signaling, Frankfurt, Germany [anti-ERK1/2 (9102), anti-phospho-ERK1/2 (9106), anti-JNK (9252), anti-phospho-JNK (9255), anti-p38 (9212), anti-phospho-p38 (9216), anti-AKT (2920), anti-phospho-AKT (4058), anti-IκBα (4814), anti-phoshpo-IκBα (2859), anti-TRAIL (3219), anti-TRAIL-R2 (3696), anti-Caspase-8 (9746), anti-Caspase-9 (9502), anti-Caspase-3 (9668), anti-PARP (9542S), anti-XIAP (2045), anti-cIAP2 (3130), anti-cIAP1 (7065), (anti-Bcl-2 (4223)]; Santa Cruz Biotechnology, Heidelberg, Germany [anti-HSP90 (sc-7947)]; BD Pharmingen, Heidelberg, Germany [anti-Bcl-xL (516,446)]; Merck Millipore [anti-TRAIL-R1 (AB16955)]; Enzo Life Sciences, Lörrach, Germany [anti-FLIP (ALX-804961)] and Sigma-Aldrich [anti-β-Actin (A5441)]. Bound primary antibodies were detected by HRP-linked secondary antibodies [Cell Signaling, anti-mouse IgG (7076) and anti-rabbit IgG (7074)] and developed in Azure Imaging System 300Q (Azure Biosystems, Dublin, California, United States) using Radiance Chemiluminescence Substrate, Radiance Q or Radiance Plus (all from Azure Biosystems) according to the manufacturer’s protocol. One representative experiment out of at least three performed is shown.

### Real-time PCR (RT-PCR)

Cells were harvested and homogenized with QIAshredder (Qiagen, Hilden, Germany) and total RNA was isolated with the RNeasy Plus Mini Kit (Qiagen) according to the manufacturer’s protocol. Complementary DNA was synthesized using the Maxima First Strand cDNA Synthesis Kit (Thermo Fisher Scientific). The expression of TRAIL-R4 was studied by RT-PCR using TaqMan assays and a 7900HT Fast RT-PCR system (all from Thermo Fisher Scientific). The expression levels were calculated relative to the expression of the housekeeping gene TATA-binding protein (TBP) by the ΔΔCT method. Primers were purchased from Thermo Fisher Scientific TBP [(Hs00427620_mL) and TRAIL-R4 (Hs00388742_mL)].

### Statistical analyses

Data are presented as mean ± SD of at least three independent experiments unless other is mentioned. Data analyses were performed using GraphPad Prism 9.0 (GraphPad Software, San Diego, California, United States). Normal distribution and equal variance of the data were assumed. For comparison of two-groups two-sided *t*-test was performed. When several groups were analyzed, one-way or two-way ANOVA with Tukey’s multiple comparison test was used. Differences between the groups were regarded statistically significant at *p*-values < 0.05 and marked with asterisks: *p < 0.05, **p < 0.01, ***p < 0.001, ****p < 0.0001.

## Results

**FIGURE 1:**
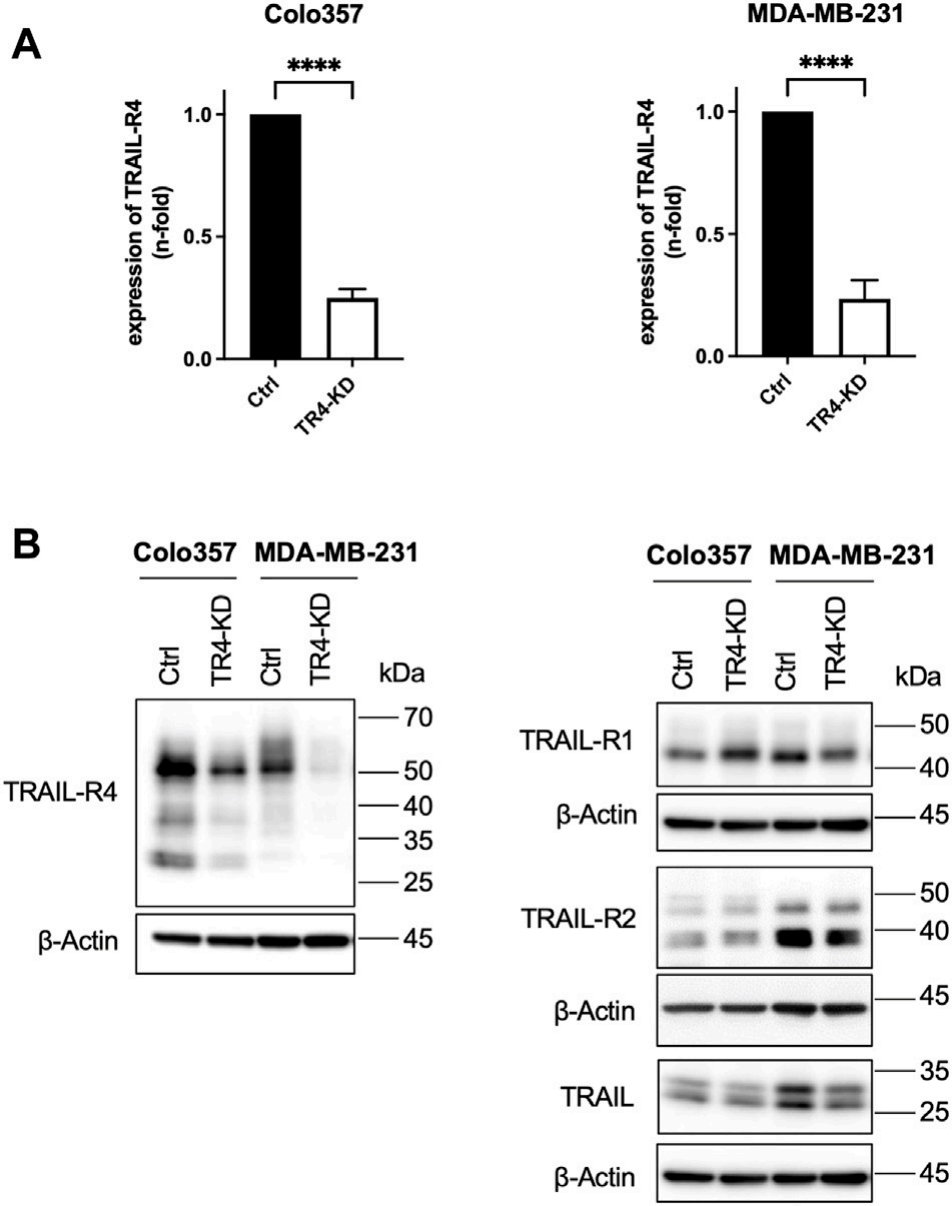
Establishment of Colo357 and MDA-MB-231 TRAIL-R4-knockdown cells. **(A)** Quantitative PCR showing the relative expression of TRAIL receptor 4 (TRAIL-R4) in Colo357 and MDA-MB-231 TRAIL-R4-knockdown clones (TR4-KD) normalized to control cells (Ctrl). Shown are the mean ± SD of three biological replicates (*n* = 3). The asterisks (****) indicate significance (*p* < 0.0001). **(B)** Western blot analysis of TRAIL-R4 expression from whole-cell lysates of Colo357 and MDA-MB-231 Ctrl and TR4-KD cells. Detection of β-Actin served as a loading control. Representative picture from at least five biological replicates.

To elaborate on the impact of the physiological levels of TRAIL-R4 on TRAIL-induced apoptotic and non-apoptotic signaling we have chosen two different cell lines, Colo357 and MDA-MB-231. These are two model cell lines for studying pancreatic cancer (Colo357) and breast cancer (MDA-MB-231), representing two human cancer entities with well-documented prognostic significance of the TRAIL/TRAIL-R system (Ganten et al., 2009; Haselmann et al., 2014; von Karstedt et al., 2015; Gundlach et al., 2018; Heilmann et al., 2019). Both cell lines express the same set of TRAIL-Rs (TRAIL-R1, -R2, and -R4 but not TRAIL-R3) (Figure 1) and have been extensively characterized with respect to their sensitivity to TRAIL by us and others (Hinz et al., 2000; Siegmund et al., 2007; Fritsche et al., 2015; Legler et al., 2018).

### Establishment of Colo357 and MDA-MB-231 TRAIL-R4-knockdown cells

To obtain cells with stable knockdown of TRAIL-R4, Colo357 and MDA-MB-231 cells were transfected with TRAIL-R4-targeting shRNA and clone pools (TRAIL-R4-KD) were established. In parallel, non-silencing shRNA-expressing clone pools (control cells) were generated. The effectiveness of knockdown of TRAIL-R4 was verified at the mRNA level and protein level by RT-PCR and Western blot analysis, respectively (Figure 1). In both cell lines, a strong reduction of TRAIL-R4 levels, both mRNA and protein, was achieved. In Colo357 cells knockdown of TRAIL-R4 resulted in slightly increased levels of TRAIL-R1, an effect which was not observed in MDA-MB-231 cells. However, in these cells, slightly diminished levels of TRAIL-R2 were observed after knocking down of TRAIL-R4. Of note, both cell lines also express TRAIL. TRAIL levels were slightly decreased by TRAIL-R4-KD in MDA-MB-231 but not in Colo357 cells.

### Knockdown of TRAIL-R4 differentially impacts on TRAIL-induced cell death in Colo357 and MDA-MB-231 cells

**FIGURE 2:**
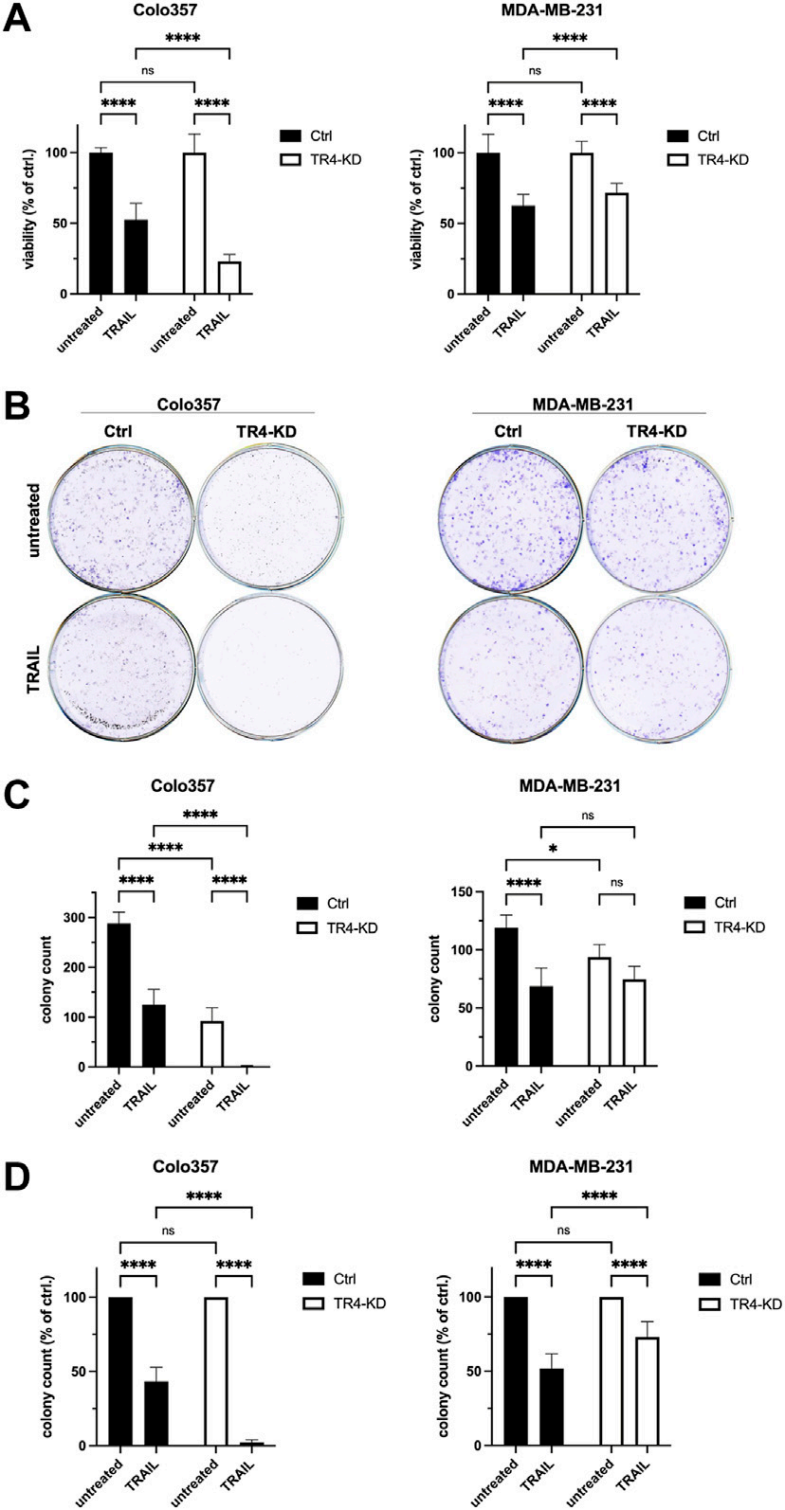
Opposite impact of TRAIL-R4 on cell viability and clonogenic survival after TRAIL treatment in Colo357 and MDA-MB-231 cells. **(A)** Viability of Colo357 and MDA-MB-231 with and without knockdown of TRAIL-R4 (TR4-KD) relative to control cells (Ctrl) analyzed by crystal violet viability assay. Cells were seeded at 1 × 10^4^ cells/well in 96-well format. After 24 h cells were stimulated with TRAIL (Colo357—50 ng/ml; MDA-MB-231—100 ng/ml) for 24 h or left untreated. Subsequently, crystal violet viability assay was performed. Shown are the mean ± SD of two (Colo357) or three (MDA-MB-231) independent experiments (*n* = 2, *n* = 3), respectively. Each experiment was performed in at least eight technical replicates. The asterisks (****/**) indicate significance (*p* < 0.0001/<0.01). **(B)** Clonogenic survival assay of untreated and TRAIL-treated Colo357 and MDA-MB-231 TR4-KD cells relative to Ctrl cells. Cells were seeded at 1 × 10^4^ cells/well in 6-well format as technical triplicates and stimulated with TRAIL (Colo357—50 ng/ml; MDA-MB-231—100 ng/ml) for 24 h, respectively or left untreated. After 6 days of further incubation without TRAIL colonies were visualized and quantified by CELLAVISTA^®^ imager and subsequently stained with crystal violet dye. Crystal violet stained colonies; shown is one representative well of each condition from one related experiment. **(C)** Total colony count per well quantified by CELLAVISTA^®^ imager. **(D)** Colony count of TRAIL-treated cells relative to untreated cells of either Ctrl or TR4-KD clones. Shown are the mean ± SD of three independent experiments (*n* = 3) performed in triplicates. The asterisks (****) indicate significance (*p* < 0.0001).

To study the impact of the endogenous TRAIL-R4 on TRAIL-induced apoptosis, TRAIL-R4-KD cells, as well as control cells, were treated with TRAIL for 24 h and cell viability was determined by crystal violet staining. As expected, the viability of control cells, both Colo357 and MDA-MB-231 cells, was clearly decreased following TRAIL treatment (Figure 2A). In line with the proposed apoptosis-protective function of TRAIL-R4, knockdown of this receptor in Colo357 strongly sensitized these cells to TRAIL, reducing their viability by 30%. Surprisingly, in MDA-MB-231 cells, TRAIL-R4 knockdown led to a rather opposite effect: instead of sensitizing, it even slightly protected these cells from TRAIL-induced cell death. To study the impact of TRAIL-R4 and TRAIL on cell proliferation and the cells’ ability to form colonies, clonogenic survival assay was performed. As shown in Figures 2B–D, knockdown of TRAIL-R4 vastly reduced the number of colonies in Colo357 (by ∼70%), while this effect was less potent but still clearly present in MDA-MB-231 cells (by ∼21%). Consistent with previous data (Legler et al., 2018), treatment with TRAIL strongly reduced clonogenic survival of Colo357 cells (by ∼57%), an effect which was also observed in MDA-MB-231 cells (by ∼43%). Importantly, knockdown of TRAIL-R4 differentially affected clonogenic survival of the studied cell lines after TRAIL treatment. In Colo357, TRAIL-R4 knockdown drastically potentiated the TRAIL effects and almost no colonies were formed in TRAIL-treated Colo357-R4-KD cells. In contrast, knockdown of TRAIL-R4 in MDA-MB-231 cells attenuated TRAIL effects, as the TRAIL-treated TRAIL-R4-KD clone formed more colonies proportionally to control cells (73.1% vs. 51.8% of colonies per well relative to their untreated controls). Thus, both, crystal violet- and clonogenic survival assay, suggest an opposite role of TRAIL-R4 in TRAIL-induced cell death in Colo357 and MDA-MB-231 cells.

**FIGURE 3:**
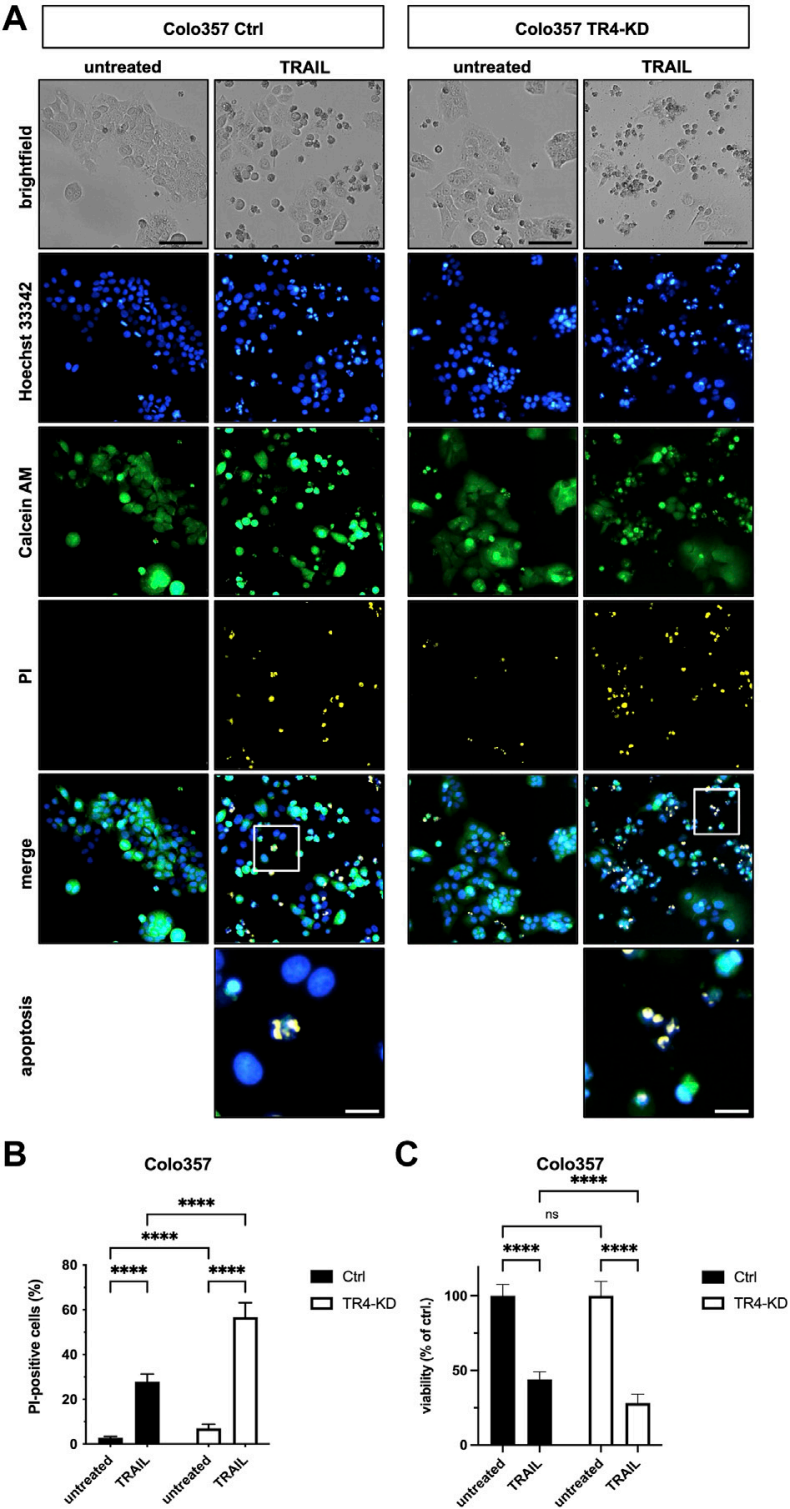
Knockdown of TRAIL-R4 increases TRAIL-mediated cell death of Colo357 cells. Colo357 control (Ctrl) and TRAIL-R4-knockdown (TR4-KD) cells were seeded at 1 × 10^4^ cells/well in 96-well format and 24 h later stimulated with 50 ng/ml TRAIL for additional 24 h or left untreated. Subsequently, cells were stained with Propidium Iodide (PI), Calcein AM and Hoechst 33342 and visualized and quantified by CELLAVISTA^®^ cell imager. **(A)** Representative pictures of the cells (brightfield) as well as Hoechst 33342 stained nuclei, Calcein AM stained cytoplasma and PI stained DNA of dead cells. The lower two pictures show representative pictures of apoptotic cells with apoptotic body formation. Black scale bars indicate 100 μm, and white scale bars indicate 20 µm. **(B)** PI-positive cells in % of all cells in untreated and TRAIL-treated Ctrl and TR4-KD cells. **(C)** Cell viability was calculated from Calcein AM and Hoechst 33342 positive cells relative to total cell count and normalized to untreated cells. Shown are the mean ± SD of four independent experiments (*n* = 4) each performed in octaplicates. The asterisks (****) indicate significance (*p* < 0.0001). **(A)** shows representative pictures from one related experiment.

**FIGURE 4:**
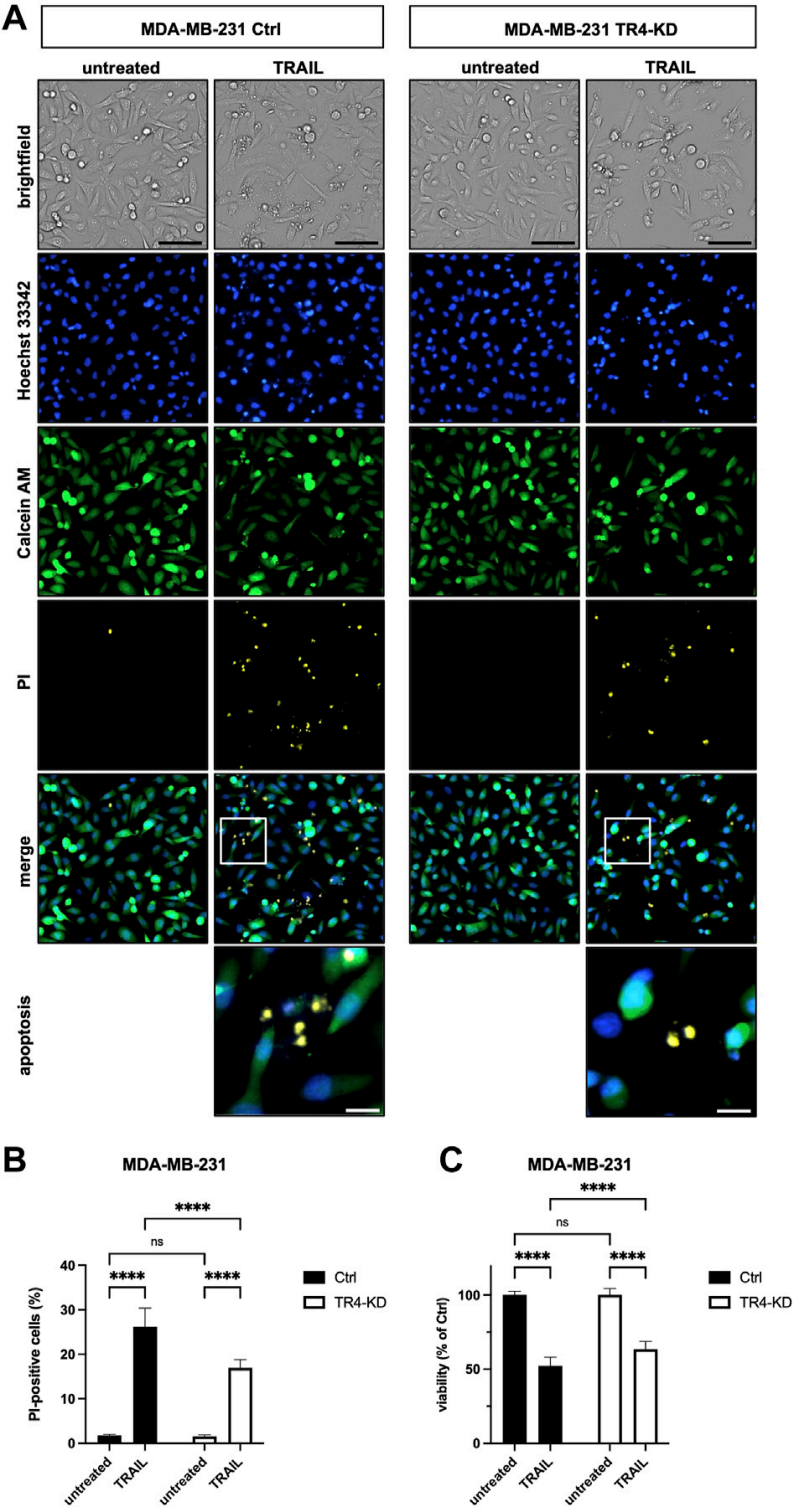
Knockdown of TRAIL-R4 impairs TRAIL-mediated cell death of MDA-MB-231 cells. MDA-MB-231 control (Ctrl) and TRAIL-R4-knockdown (TR4-KD) cells were seeded at 1 × 10^4^ cells/well in 96-well format and 24 h later stimulated with 100 ng/ml TRAIL for additional 24 h or left untreated. Subsequently, cells were stained with Propidium Iodide (PI), Calcein AM and Hoechst 33342 and visualized and quantified by CELLAVISTA^®^ cell imager. **(A)** Representative pictures of the cells (brightfield), as well as, Hoechst 33342 stained nuclei, Calcein stained cytoplasma and PI stained DNA of dead cells. The lower two pictures show representative pictures of apoptotic cells with apoptotic body formation. Black scale bars indicate 100 μm, and white scale bars indicate 20 µm. **(B)** PI-positive cells in % of all cells in untreated and TRAIL-treated Ctrl and TR4-KD cells. **(C)** Cell viability was calculated from Calcein AM and Hoechst 33342 positive cells relative to total cell count and normalized to untreated cells. Shown are the mean ± SD of four independent experiments (*n* = 4) each performed in octaplicates. The asterisks (****) indicate significance (*p* < 0.0001). (A) shows representative pictures from one related experiment.

To verify these results by an additional independent method, subsequent to TRAIL treatment, cells were stained with Calcein AM, Hoechst 33342 and propidium iodide (PI), and live and dead cells were visualized and quantified by CELLAVISTA^®^ cell imager (Colo357 Figure 3, MDA-MB-231 Figure 4). TRAIL treatment led to a diminished number of viable cells in both control cell lines (Colo357 44% viability and MDA-MB-231 52% viability, respectively), an effect which can be attributed to TRAIL-mediated cell death as revealed by PI staining. Importantly, consistent with the crystal violet- and clonogenic survival assay data, knockdown of TRAIL-R4 again had an opposite effect on TRAIL sensitivity of these cell lines. While Colo357 became clearly more TRAIL-sensitive after TRAIL-R4 knockdown (Figure 3), MDA-MB-231 cells became significantly more resistant (Figure 4).

**FIGURE 5:**
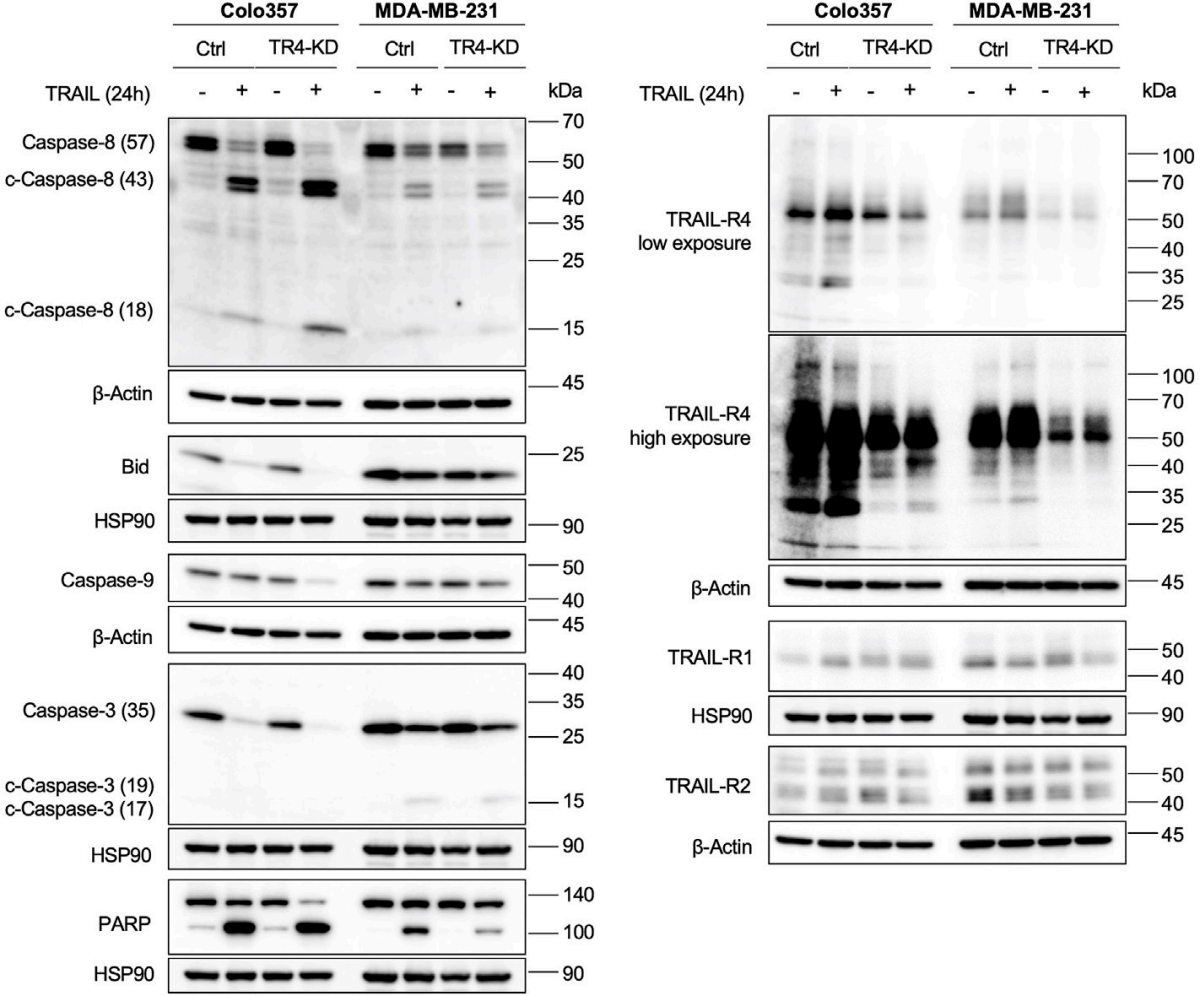
Knockdown of TRAIL-R4 impacts TRAIL-mediated apoptosis of Colo357 and MDA-MB-231 cells in an opposing manner. Colo357 and MDA-MB-231 control (Ctrl) and TRAIL-R4-knockdown (TR4-KD) cells were seeded at 3,3 × 10^5^/well (Colo357) and 3 × 10^5^/well (MDA-MB-231) in 6-well format and 24 h later were stimulated with TRAIL (Colo357–50 ng/ml; MDA-MB-231—100 ng/ml) for 24 h or left untreated. Whole-cell lysates were analyzed by Western blot for the levels of Caspase-8, Bid, Caspase-9, Caspase-3 and PARP. Detection of either β-Actin or HSP90 served as a gel loading control. Shown are representative pictures from one related experiment out of at least three biological replicates.

To further confirm these observations, we studied the effects of TRAIL-R4 knockdown on the TRAIL-mediated activation of the apoptosis signaling pathway by Western blot analysis performed side by side for both cell lines. In accordance with the cell death and viability quantifying data, the results shown in Figure 5 substantiated the apoptosis-sensitizing effects of TRAIL-R4 knockdown in Colo357 cells and obviously protective effects thereof in MDA-MB-231 cells. Concretely, in Colo357 cells, all analyzed steps of the apoptotic pathway: cleavage of Caspase-8, Bid, Caspase-9, Caspase-3 and finally the cleavage of the Caspase-3 target PARP documented a stronger activation of the pathway in TRAIL-R4-KD than in corresponding control cells. In contrast, PARP cleavage after TRAIL treatment was less pronounced in MDA-MB-231 TRAIL-R4-KD than in control cells.

Altogether, these data suggest that TRAIL-R4 might differentially regulate TRAIL-induced apoptosis, acting either protective or sensitizing in a cell-dependent manner.

### Knockdown of TRAIL-R4 shows opposite effects on the non-canonical TRAIL-induced signal transduction pathways in Colo357 and MDA-MB-231 cells

It is well known that, besides apoptosis, TRAIL, via triggering of its death receptors TRAIL-R1 and TRAIL-R2, can also induce several non-canonical, pro-inflammatory signal transduction pathways (Azijli et al., 2013). To the best of our knowledge, the impact of endogenous TRAIL-R4 on these TRAIL-induced pathways is unknown so far.

To study these pathways, control cells, as well as TRAIL-R4-KD cells, were stimulated with TRAIL for 3 h, and subsequently the phosphorylation/activity of AKT, MAPKs, as well as the phosphorylation of IκBα as an indicator for the activation of NF-κB were analyzed by Western blotting using phospho-specific antibodies. As a control, the overall cellular expression levels of these proteins were analyzed in parallel.

**FIGURE 6:**
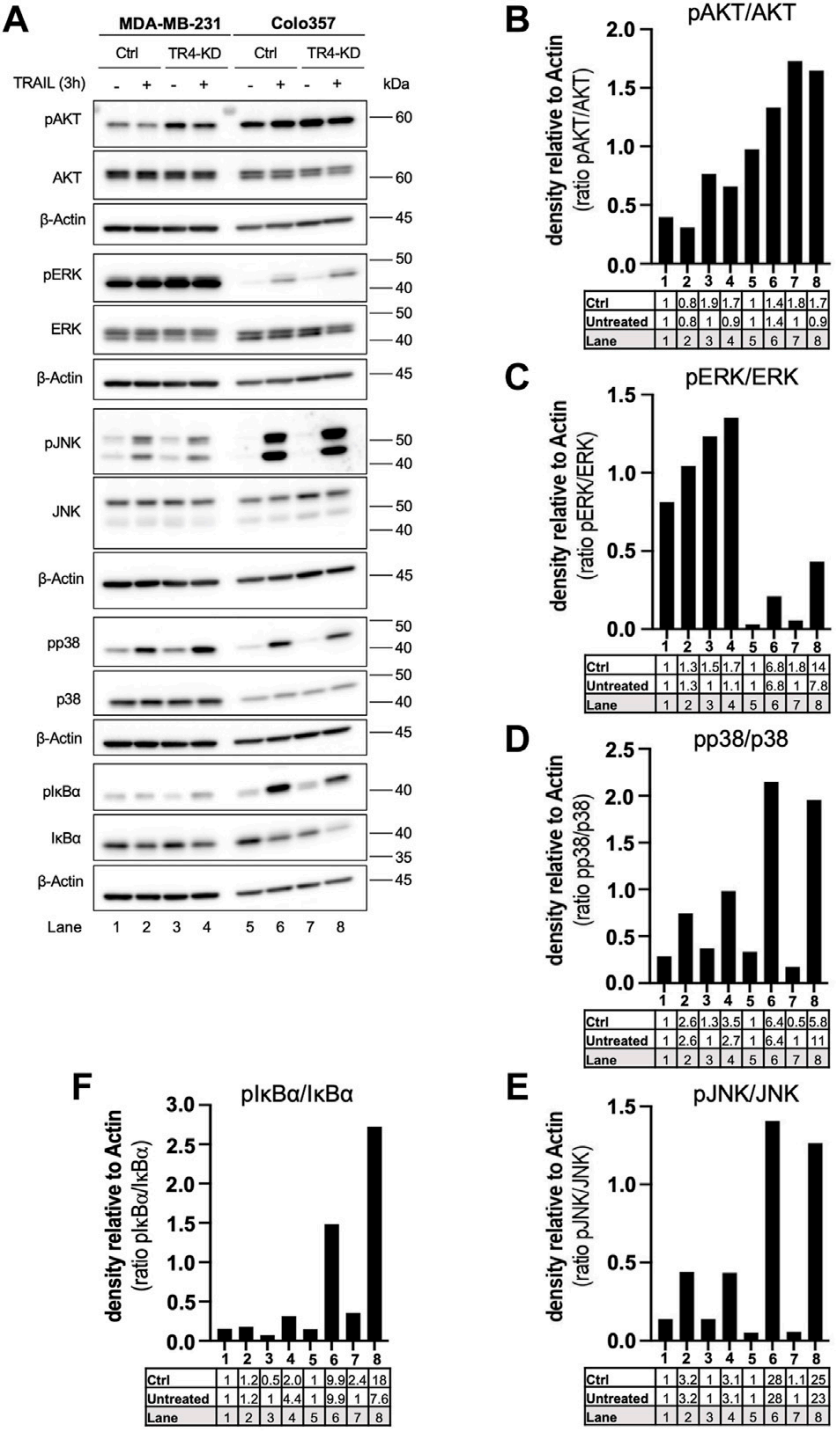
Impact of TRAIL-R4 on TRAIL-mediated non-apoptotic signaling in Colo357 and MDA-MB-231 cells. Colo357 and MDA-MB-231 control (Ctrl) and TRAIL-R4-knockdown (TR4-KD) cells were seeded at 3.3 × 10^5^/well (Colo357) and 3 × 10^5^/well (MDA-MB-231) in 6-well format and 24 h later were stimulated with TRAIL (Colo357—50 ng/ml; MDA-MB-231—100 ng/ml) for 24 h or left untreated. **(A)** Whole-cell lysates were analyzed by Western blot for the levels of pAKT/AKT, pERK/ERK, pJNK/JNK, pp38/p38 and pIκBα/IκBα. Detection of either β-Actin or HSP90 served as a gel loading control. Shown are representative pictures from one related experiment out of at least three biological replicates. **(B–F)** The ratios of pAKT/AKT, pERK/ERK, pJNK/JNK, pp38/p38 and pIκBα/IκBα were analyzed by densitometry. Intensity of each band was normalized to β-Actin.

Comparison of TRAIL treated cells with the respective untreated cells revealed that in both parental cell lines, TRAIL treatment resulted in the activation of ERK, JNK, p38 and NF-κB (Figure 6). All these effects were clearly more pronounced in Colo357 than in MDA-MB-231 cells. Interestingly, the knockdown of TRAIL-R4 differentially affected these pathways in the cell lines. While TRAIL-R4 knockdown improved the capacity of TRAIL to activate p38 and ERK in Colo357 cells, no such effects were detected in MDA-MB-231 cells. Concerning the involvement of TRAIL-R4 in TRAIL-induced NF-κB activation, opposite effects were observed compared to MAPKs. In this case knockdown of TRAIL-R4 markedly enhanced TRAIL-induced phosphorylation of IκBα in MDA-MB-231 whereas it rather inhibited this pathway in TRAIL-treated Colo357 cells. In addition, in Colo357 control cells, TRAIL activated AKT while no activation but even decreased levels of active AKT were detected in TRAIL-stimulated Colo357 TRAIL-R4-KD cells. In MDA-MB-231 cells, in both control cells as well as in TRAIL-R4-KD cells treatment with TRAIL slightly decreased the levels of active AKT. Importantly, in both cell lines the activity of AKT, ERK, p38 and NF-κB after TRAIL treatment was higher in TRAIL-R4 KD cells than in respective TRAIL-treated control cells.

Intriguingly, consistent in both cell lines, knockdown of TRAIL-R4 resulted in constitutively increased activity of AKT and ERK. Of note, MDA-MB-231 cells showed overall much higher ERK activity than Colo357 cells. On the contrary, the overall activity of AKT was higher in Colo357 compared to MDA-MB-231 cells.

### Impact of TRAIL-R4 on the expression levels of anti-apoptotic proteins, TRAIL-Rs and TRAIL in MDA-MB-231 and Colo357 cells

**FIGURE 7:**
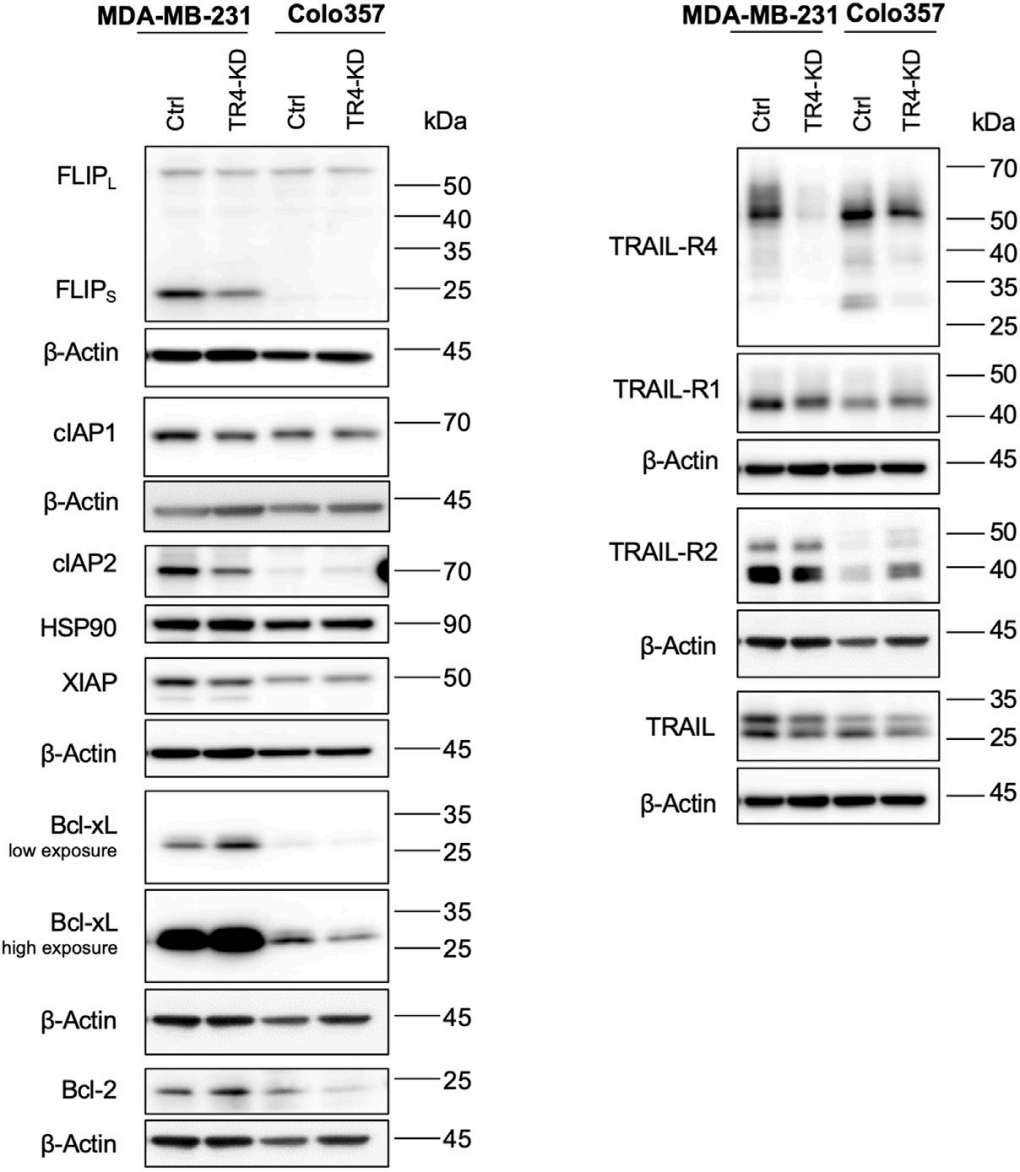
Impact of TRAIL-R4 on the expression levels of anti-apoptotic proteins, TRAIL-Rs and TRAIL in MDA-MB-231 and Colo357 cells. MDA-MB-231 and Colo357 control (Ctrl) and TRAIL-R4-knockdown (TR4-KD) cells were seeded at 3.3 × 10^5^/well (Colo357) and 3 × 10^5^/well (MDA-MB-231) in 6-well format and 24 h later whole-cell lysates were prepared. Western blot analysis of anti-apoptotic proteins FLIPL/S, cIAP1, cIAP2, XIAP, Bcl-xL, Bcl-2 and TRAIL receptors (TRAIL-R4, -R1, -R2) and TRAIL were performed. Detection of β-Actin served as a loading control. Shown are representative pictures from one related experiment out of three biological replicates.

Constitutive and concomitant upregulation of different anti-apoptotic proteins is a known hallmark of cancer (Hanahan and Weinberg, 2000). Colo357 and MDA-MB-231 cells are both type II cells, which as such require the mitochondrial amplification loop for efficient apoptosis induction. Thus, in these cells, apoptosis can be inhibited at the receptor-, mitochondria- and/or caspase activity level. To study whether TRAIL-R4 influences the expression of the main regulators of TRAIL-mediated apoptosis, the expression levels of FLIP, Bcl-xL, Bcl-2, cIAP1, cIAP2 and XIAP were analyzed side by side in Colo357 and MDA-MB-231 cells with and without TRAIL-R4 knockdown. The expression of TRAIL-Rs and TRAIL was analyzed in parallel (Figure 7).

Comparing Colo357 and MDA-MB-231 control cells, the latter clearly expressed much higher levels of anti-apoptotic proteins, especially FLIPs, Bcl-xL, XIAP and cIAP2. Interestingly, also the levels of both TRAIL death receptors, particularly TRAIL-R2, as well as the levels of TRAIL were higher in MDA-MB-231 than in Colo357 cells.

Moreover, TRAIL-R4 turned out to be an important regulator of the expression of almost all anti-apoptotic proteins in MDA-MB-231 cells, influencing their cellular levels either positively or negatively. In terms of fact, while the levels of FLIPs, cIAP2 and XIAP decreased in TRAIL-R4-KD compared to control cells, the levels of Bcl-xL significantly increased. In Colo357, the knockdown of TRAIL-R4 did not affect the levels of FLIPs, cIAP2, XIAP and Bcl-2. However, and in contrast to MDA-MB-231 cells, it led to the downregulation of the levels of Bcl-xL.

### Inhibition of Bcl-xL but not of Bcl-2, MEK1/2 or AKT, abolishes the apoptosis resistance of MDA-MB-231 TRAIL-R4 knockdown cells

Our data revealed that TRAIL-R4 knockdown in MDA-MB-231 cells resulted in a constitutive upregulation of two signal transduction pathways, MAPK/ERK and AKT, and an upregulation of Bcl-xL levels. Since all these effects could potentially account for the observed more apoptosis-resistant phenotype of TRAIL-R4-KD compared to control cells, we hypothesized that the inhibition of these pathways or of Bcl-xL could reverse this phenotype.

To prove the role of MAPK/ERK and AKT pathways, cells were pre-treated with pharmacological inhibitors of these pathways, U0126 and MK-2206, respectively, and subsequently exposed to TRAIL for 24 h. U0126 is an inhibitor of MEK1/2 kinases which are responsible for the activation of ERK1/2 whereas MK-2206 is a highly selective inhibitor of AKT1/2/3. Following treatment, cell viability was studied by crystal violet staining.

**FIGURE 8:**
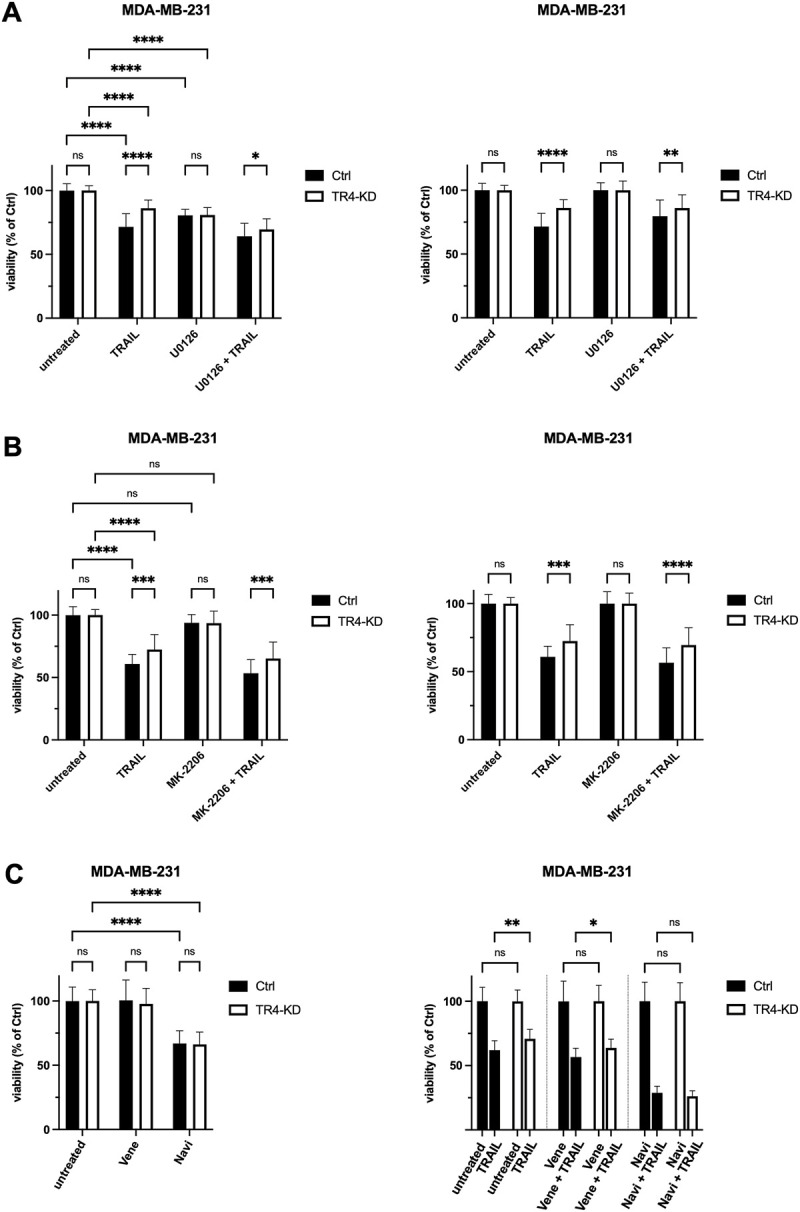
Inhibition of Bcl-xL but not of Bcl-2, MEK1/2 or AKT, abolishes the apoptosis resistance of MDA-MB-231 TRAIL-R4 knockdown cells. Crystal violet viability assay after treatment with pharmacological inhibitors. MDA-MB-231 control (Ctrl) and TRAIL-R4-knockdown (TR4-KD) cells were seeded at 1 × 10^4^ cells/well in 96-well format and pre-treated with **(A)** MEK1/2 inhibitor (U0126; 10 µM), **(B)** AKT inhibitor (MK-2206; 1 µM), **(C)** Bcl-2 inhibitor (Venetoclax (Vene); 5 µM), Bcl-2/Bcl-xL inhibitor (Navitoclax (Navi); 5 µM) or specific solvents as controls. After 2 h TRAIL was added at 50 ng/ml (Colo357) or 100 ng/ml (MDA-MB-231) for additional 24 h. Subsequently, cell viability was analyzed by crystal violet viability assay. Shown are the mean ± SD of four biological replicates (*n* = 4) for experiments with U0126 and MK-2206 and three biological replicates (*n* = 3) for experiments with Navitoclax and Venetoclax, respectively. All experiments were performed in six technical replicates. The asterisks (*/**/****) indicate significance (*p* < 0.05/< 0.01/< 0.0001).

As shown before, TRAIL-R4-KD cells displayed a significantly decreased sensitivity to TRAIL compared to control cells (Figures 8A,B). Neither pre-treatment with MEK- nor with AKT-inhibitor changed the relative difference in the viability of TRAIL-R4-KD vs. control cells (Figures 8A,B).

To investigate the possible involvement of Bcl-xL in the observed diminished sensitivity of MDA-MB-231 TRAIL-R4-KD cells compared to the parental cells, we used the pharmacologic inhibitors Navitoclax (ABT-263) and Venetoclax (ABT-199). Navitoclax potently antagonizes Bcl-2 and Bcl-xL, whereas Venetoclax selectively inhibits Bcl-2. MDA-MB-231 control and TRAIL-R4-KD cells were pre-treated with inhibitors for 2 h prior to treatment with TRAIL for additional 24 h. Cell viability was analyzed by crystal violet staining. While Venetoclax only marginally affected cell viability of both control and TRAIL-R4-KD cells (mean viability 100% and 97.7%), treatment with Navitoclax alone already led to a reduction in cell viability by 33% in both cell lines (Figure 8C). Interestingly, while the combination of Venetoclax plus TRAIL did not affect the ratio of viability between control and TRAIL-R4-KD cells in favor of higher viability of the TRAIL-R4-KD cells (mean viability 56.6% vs. 63.7%, *p* = 0.0187, Figure 8C), the combination of Navitoclax plus TRAIL abolished the apoptosis resistance of MDA-MB-231 TRAIL-R4-KD cells, shown in equal viability of control and TRAIL-R4-KD cells (mean viability 28.8% vs. 26.2%, *p* = 0.982, Figure 8C). These results underline the decisive role of Bcl-xL for apoptosis resistance of MDA-MB-231 in general. Moreover, these findings indicate that the upregulated expression of Bcl-xL in MDA-MB-231 TRAIL-R4-KD cells is, at least partly, responsible for the unexpected TRAIL-resistant phenotype of these TRAIL-R4-KD cells.

### Inhibition of IAPs dramatically reduces the viability of MDA-MB-231 TRAIL-R4-KD cells

Our data revealed that, besides Bcl-xL, MDA-MB-231 cells express high levels of cIAP1, cIAP2 and XIAP. Although they are down regulated in TRAIL-R4-KD cells, the levels of cIAP2 and XIAP are still much higher in these cells than in Colo357 cells. Since IAPs are known inhibitors of TRAIL-induced cell death, we wondered whether they influence the sensitivity of MDA-MB-231 control and TRAIL-R4 KD cells to TRAIL.

**FIGURE 9:**
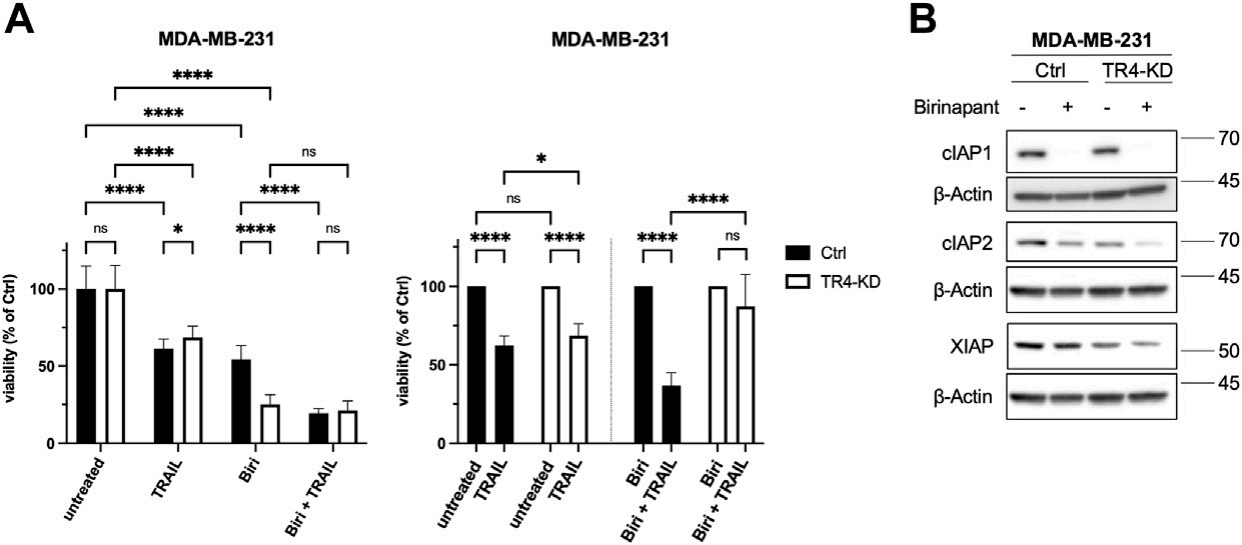
Inhibition of IAPs drastically reduces the viability of MDA-MB-231 TRAIL-R4-KD cells. **(A)** MDA-MB-231 control (Ctrl) and TRAIL-R4-knockdown (TR4-KD) cells were treated with either TRAIL (100 ng/ml) or Birinapant (1 μM; Biri), or combination of both. Viability of cells was studied 24 h after TRAIL addition by crystal violet viability assay. Shown are the mean ± SD of 3 biological replicates (*n* = 3). All experiments were performed in 12 technical replicates. The asterisks (*/**/****) indicate significance (*p* < 0.05/< 0.01/< 0.0001). **(B)** Western blot showing effects of Birinapant treatment (1 μM, 6 h) on the levels of cIAP1, cIAP2 and XIAP in MDA-MB-231 cells with and without TRAIL-R4-KD. Detection of β-Actin served as a loading control. Shown are representative pictures from one related experiment out of four biological replicates.

To determine the impact of IAPs on the sensitivity of MDA-MB-231 cells to TRAIL, cells were pre-treated with Birinapant prior to their exposition to TRAIL for 24 h. Cell viability was analyzed by crystal violet staining. Birinapant (TL32711), a bivalent SMAC mimetic acts as a potent inhibitor of cIAP1, cIAP2 and XIAP and is currently tested in clinical trials (Morrish et al., 2020). Binding of Birinapant to cIAP1 and cIAP2 induces their autoubiquitylation and degradation whereas its binding to XIAP prevents its ability to bind to and inhibit the apoptotic caspases Caspase-3, -7 and Caspase-9. As shown in Figure 9B, Birinapant led to rapid degradation of cIAP1 and less effectively but still strongly down regulated the levels of cIAP2. No changes in the levels of XIAP have been detected. Consistent with previous report (Lalaoui et al., 2020), Birinapant as a single agent strongly reduced the viability of MDA-MB-231 control cells (mean viability 54.4%). Intriguingly, this effect was much more pronounced in TRAIL-R4-KD cells where only 25.2% of cells survived Birinapant treatment (Figure 9A left panel). Of note, while in control cells Birinapant and TRAIL showed additive effects, no further decrease of cell viability by TRAIL could be observed in Birinapant-treated TRAIL-R4-KD cells.

### Inhibition of MEK1/2 leads to a strong decrease of Bcl-xL in MDA-MB-231 cells

**FIGURE 10:**
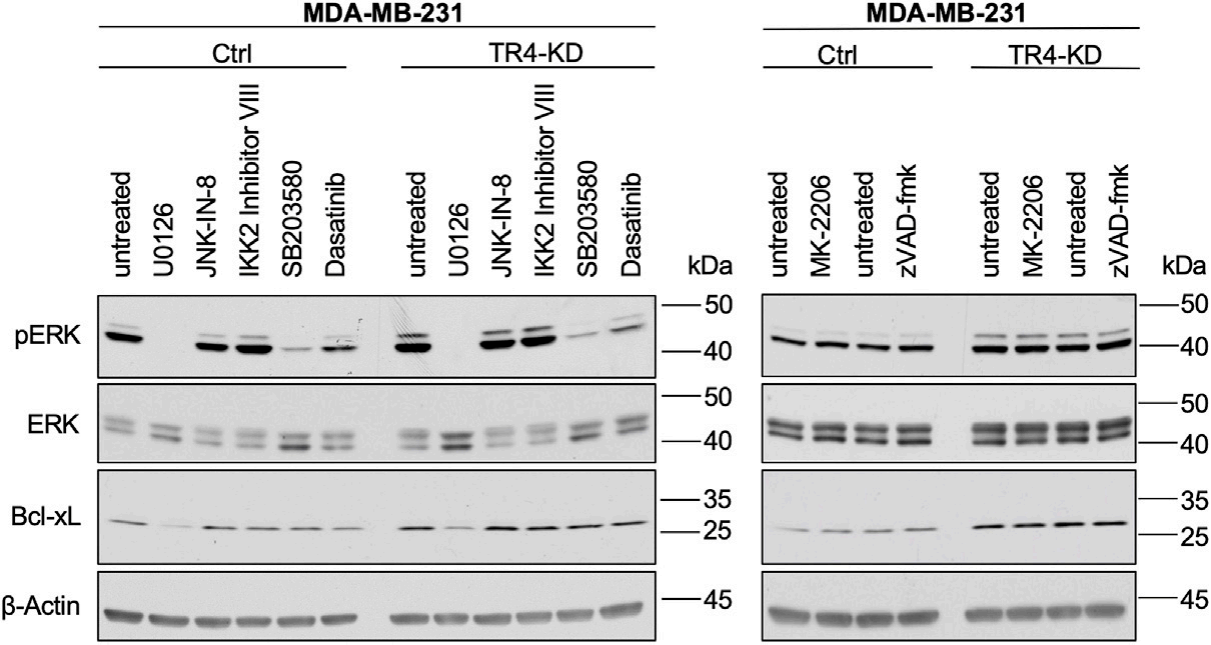
Inhibition of MEK1/2 leads to a strong decrease of Bcl-xL in MDA-MB-231 cells. MDA-MB-231 control (Ctrl) and TRAIL-R4-knockdown (TR4-KD) cells were seeded at 2–4 × 10^6^ cells/well in 6-well format and 24 h later stimulated for 2 h with pharmacological inhibitors of different signal transduction pathways at the indicated concentrations: MEK1/2 (U0126; 10 µM), JNK (JNK-IN-8; 1 µM), IKK2 (IKK2 inhibitor VIII; 10 µM), p38 (SB203580; 10 µM), Src (Dasatinib; 100 nM), AKT (MK-2206; 1 µM), broad-spectrum caspase inhibitor (zVAD-fmk; 20 µM) or specific solvents as controls. Whole-cell lysates were analyzed by Western blot for the levels of pERK, ERK and Bcl-xL. Detection of β-Actin served as a loading control. Shown are representative pictures from one related experiment out of three biological replicates.

To get insights into the mechanisms of the TRAIL-R4-mediated regulation of Bcl-xL levels, we finally asked which signal transduction pathway could be responsible for the observed high expression of Bcl-xL in MDA-MB-231 cells and its further upregulation by TRAIL-R4 knockdown. For this purpose, cells were treated with the pharmacological inhibitors of the main regulators of the pathways known to be activated by TRAIL-Rs like MEK (U0126), JNK (JNK-IN-8), IKK2 (IKK2-Inhibitor VIII), p38 (SB203580), Src (Dasatinib), AKT (MK-2206), as well as with a broad-spectrum inhibitor of apoptotic caspases (zVAD-fmk). As shown in Figure 10, inhibition of only one of these pathways, namely the MAPK/ERK pathway, led to a strong decrease of Bcl-xL levels in both, Ctrl and TRAIL-R4-KD cells.

Summing up, our results provide evidence that endogenous TRAIL-R4 plays a critical role in regulating TRAIL-induced apoptotic and non-apoptotic signaling in cancer cells and impacts the outcome of the response to TRAIL in a cell-dependent manner.

## Discussion

Almost 25 years ago, three groups independently reported the identification of a new TRAIL receptor, TRAIL-R4 (DcR2/TRUNDD), by means of its sequence homology to TRAIL-R1, TRAIL-R2 and TRAIL-R3, which were discovered only a few months before (Degli-Esposti et al., 1997a; Marsters et al., 1997; Pan et al., 1998a; Danish et al., 2017). Soon after their discovery, TRAIL-R1 and TRAIL-R2 became intensively investigated due to their potential role as targets for apoptosis-inducing anti-cancer therapy (Ashkenazi et al., 1999; Walczak et al., 1999). Meanwhile, the death-inducing as well as pro-inflammatory signaling capacities of these receptors are pretty well understood (von Karstedt et al., 2017).

The contribution of endogenous TRAIL-R4 to TRAIL-induced signaling in cancer cells is still obscure. In fact, most of the existing data addressing the functional relevance of this receptor arise from overexpression studies which “overload” the system, potentially resulting in overestimation or even confounding effects. Overexpression studies showed that TRAIL-R4 can protect tumor cells from TRAIL-induced apoptosis by acting as a decoy receptor for ligand binding and/or inhibiting DISC formation by interacting with the TRAIL death receptors (Pan et al., 1997a; Marsters et al., 1997; Clancy et al., 2005; Merino et al., 2006; Neumann et al., 2014). In addition, ectopic overexpression of TRAIL-R4 resulted in activation of NF-κB (Degli-Esposti et al., 1997a) and AKT (Lalaoui et al., 2011), demonstrating the capability of the intracellular part of TRAIL-R4 to induce these pathways, at least when highly expressed.

Recently, the relevance of endogenous TRAIL-R4 levels for the protection against TRAIL-induced cell death has been finally demonstrated (Singh et al., 2017; Tawfik et al., 2019). To the best of our knowledge, no reports have yet analyzed the impact of endogenous TRAIL-R4 levels on apoptotic and non-apoptotic TRAIL-induced signaling in cancer cells side by side.

In the present study, we show that the knockdown of endogenous TRAIL-R4 affects both apoptotic and non-apoptotic TRAIL-induced signaling. However, TRAIL-R4-mediated effects differ depending on the cell line studied, partly even showing opposite effects. Thus, concerning the apoptotic pathway, we found that TRAIL-R4 knockdown in Colo357 cells strongly sensitized these cells to TRAIL-mediated apoptosis, an effect which is in agreement with the already mentioned published data (see above). In contrast, reducing the levels of endogenous TRAIL-R4 in MDA-MB-231 cells resulted in the inhibition of TRAIL-induced apoptosis. This effect was rather unexpected and has not been described so far. Analyses of the expression levels of anti-apoptotic proteins revealed markedly upregulated Bcl-xL expression levels in MDA-MB-231 TRAIL-R4-KD compared to control cells. A rather inverse effect was observed in Colo357 cells. In line with already published data, Colo357 cells express very low levels of this protein, anyway (Hinz et al., 2000; Trauzold et al., 2003). Importantly, inhibition of Bcl-xL and Bcl-2 by Navitoclax, reversed the apoptosis-resistant phenotype of MDA-MB-231 TRAIL-R4-KD cells. Of note, the specific inhibition of Bcl-2 by Venetoclax did not change this phenotype, arguing for the importance of Bcl-xL in this context.

In contrast to the upregulation of Bcl-xL, knockdown of TRAIL-R4 clearly reduced the expression levels of FLIP, XIAP and cIAP2 in MDA-MB-231 cells. Although the mechanism responsible for these effects remains to be elucidated, it is likely that the decreased NF-κB activity accounts for this phenomenon, since TRAIL-R4-KD cells show reduced levels of pIκBα.

Intriguingly, the viability of MDA-MB-231 TRAIL-R4-KD cells was dramatically impaired by the inhibition of IAPs, much more than of the respective control cells. The mechanisms behind these effects remain to be elucidated. However, it seems possible that endogenous TRAIL or other death inducing ligands like TNFα or CD95L could account for these effects. Such Birinapant-induced, endogenous TNFα- and TRAIL-dependent cell death has already been described for different tumor cells, among them also for triple negative breast cancer cells (TNBC) (Petersen et al., 2007; Varfolomeev et al., 2007; Lalaoui et al., 2020). Interestingly, Birinapant did not affect the viability of Colo357 cells which compared to MDA-MB-231 cells expressed lower levels of cIAP2 and XIAP and comparable levels of cIAP1. Neither the control cells nor TRAIL-R4-KD cells were significantly affected by Birinapant treatment (data not shown). Why the killing potential of Birinapant as a single agent is potentiated by knockdown of TRAIL-R4 in MDA-MB 231 cells but not in Colo357 cells, is not known and needs to be clarified by further studies. Recently, the predictive signature for the responsiveness of malignant melanoma cells to the combination of Birinapant and the latest generation hexavalent TRAIL-based biologic IZI1551 was generated (Vetma et al., 2020). It has been proposed, that a combination of Caspase-3, XIAP and the members of Bcl-2 family together with the non-limited TRAIL death receptors and Caspase-8 play a crucial role in the sensitivity to IZI1551/Birinapant treatment. Since MDA-MB-231 and Colo357 cells significantly differ in the expression levels of these molecules while both express endogenous TRAIL, it is possible that similar mechanisms could account for the observed differences in the sensitivity of these cells to Birinapant. Future studies with other cell lines expressing endogenous TRAIL would help to clarify this issue.

Given that TRAIL plays an important role in immune tumor surveillance (Falschlehner et al., 2009) and that TRAIL-R4 can act as an inhibitor of TRAIL-mediated cell death, it could be assumed that TRAIL-R4 should be highly expressed in malignant cells as self-protection mechanism. This is partly true. In some cancer entities, immunohistochemical studies showed a correlation between TRAIL-R4, disease progression and poor patients’ prognosis, suggesting a tumor-promoting role of high TRAIL-R4 levels (Koksal et al., 2008; Ganten et al., 2009; Sanlioglu et al., 2009).

However, it was also reported that the TRAIL-R4 gene is highly methylated in many tumors including melanoma, neuroblastoma, pheochromocytoma, breast and lung cancer. Additionally, some recent studies indicated that the methylation of the TRAIL-R4 promoter indeed predisposes to tumor formation, relapse and overall poor prognosis (van Noesel et al., 2002; Shivapurkar et al., 2004; Yang et al., 2007; Bonazzi et al., 2011; Lau et al., 2012; Ratzinger et al., 2014). Therefore, one can deduce that TRAIL-R4 bears more functions than currently recognized. It is also conceivable, that TRAIL-R4 acts differently, either increasing or inhibiting tumor malignancy, depending on the cellular context. In this regard, we have recently found that knockdown of TRAIL-R4 in Colo357 cells inhibits the killing potential of γδ T-cells (Tawfik et al., 2019). The mechanism behind this phenomenon could be attributed to the upregulation of ERK-activity in TRAIL-R4-KD cells, resulting in an enhanced expression of COX2 which in turn inhibits the γδ T-cell killing activity.

In the present study, we show that knockdown of TRAIL-R4 increased ERK-activity in MDA-MB-231 cells as well, leading to the upregulation of Bcl-xL and decreased sensitivity of these cells to treatment with recombinant TRAIL. Constitutive upregulation of Bcl-xL is frequently observed in cancer and plays a decisive role for the protection against intrinsic apoptosis and extrinsic apoptosis in type II cells, respectively (Scaffidi et al., 1998).

Interestingly, in both cell lines not only ERK- but also AKT-activity was strongly upregulated by knockdown of TRAIL-R4, suggesting a common mechanism behind these effects. Both, ERK and AKT are key proteins of signaling pathways known to be induced by TRAIL death receptors. Since both cell lines express TRAIL, it is likely that in TRAIL-R4-KD cells binding of tumor cell-derived TRAIL to TRAIL-R1/R2 results in the constitutively increased activity of ERK and AKT. Such endogenous TRAIL-dependent TRAIL-R2-mediated activation of the Rac1/PI3K/AKT pathway has been shown to promote migration, invasion and metastasis of KRAS-mutated cancer cells (von Karstedt et al., 2015). Of note, both Colo357 and MDA-MB-231 cells are KRAS-mutated cell lines, thus prone to the activation of this pathway by endogenous TRAIL. The upregulation of AKT-activity by TRAIL-R4 knockdown strongly indicates that this receptor might act as an endogenous negative regulator of this cancer cell-autonomous malignancy-promoting TRAIL-R signaling pathway. Interestingly, whereas knockdown of TRAIL-R4 in both cell lines led to an increase in the constitutive activity of AKT, it resulted in a decrease of AKT-activity after treatment with recombinant TRAIL. This suggests that endogenous and recombinant TRAIL might engage different receptors or signaling complexes to induce the Rac1/PI3K/AKT pathway.

Regarding the involvement of TRAIL-R4 in the response of Colo357 cells to exogenous TRAIL, two different effects with potentially opposite consequences for tumor (patho-) physiology were detected. While, following TRAIL-treatment the activation of apoptotic caspases was significantly upregulated in TRAIL-R4-KD cells, also the activity level of pro-inflammatory signaling pathways, including ERK AKT, p38 and NF-κB was clearly enhanced. The former rather indicates anti-tumoral functions, the latter pro-tumoral effects. Previously, we have shown that TRAIL-induced activation of ERK, JNK and NF-κB requires the activity of apoptotic caspases in Colo357 cells (Siegmund et al., 2007). Importantly, these pathways are involved in the TRAIL-mediated induction of pro-inflammatory cytokines which in turn promote tumor cell migration, invasion and metastasis. Besides affecting the tumor cells, themselves, the TRAIL-induced cancer cell secretome has been shown to promote tumor malignancy by inducing the accumulation of tumor-supporting immune cells in the tumor microenvironment. This effect has been attributed to TRAIL death receptors and requires their DD, recruitment of FADD and the presence of Caspase-8 but not its activity (Hartwig et al., 2017).

Further investigations are needed to figure out whether the upregulation of the activity of pro-inflammatory pathways in TRAIL-treated Colo357 TRAIL-R4-KD cells results in enhanced cytokine production and thereby increases malignancy. This, in turn, would attribute an anti-tumoral function to the presence of TRAIL-R4 in these cancer cells.

Summing up, we found that TRAIL-R4 exerts different, partly opposing functions particularly concerning the cells’ sensitivity to TRAIL-mediated apoptosis in human cancer cell lines Colo357 and MDA-MB-231 depending on their constitutive repertoire of non- and anti-apoptotic protein levels. Furthermore, we here show for the first time that the presence of TRAIL-R4 itself decisively affects the expression levels of several key proteins of pro-inflammatory and anti-apoptotic signal transduction pathways and regulates exogenous TRAIL-mediated non-apoptotic signaling. Our study improves the understanding of the TRAIL/TRAIL-R system and points to the necessity of further studies addressing the functions of TRAIL-R4 and endogenous TRAIL in cancer. This may open new opportunities for targeted therapeutic interventions in the future.

## Data Availability

The raw data supporting the conclusions of this article will be made available by the authors, without undue reservation.
